# Fly-By medical care: Conceptualizing the global and local social responsibilities of medical tourists and physician voluntourists

**DOI:** 10.1186/1744-8603-7-6

**Published:** 2011-04-06

**Authors:** Jeremy Snyder, Shafik Dharamsi, Valorie A Crooks

**Affiliations:** 1 Faculty of Health Sciences, Simon Fraser University, Blusson Hall 11300, 8888 University Drive Burnaby BC, Canada; 2 Department of Family Practice, University of British Columbia, David Strangway Building, 3rd Floor 5950 University Boulevard, Vancouver, BC, Canada; 3 Department of Geography, RCB 6141, Simon Fraser University 8888 University Drive Burnaby, B C, Canada

## Abstract

**Background:**

Medical tourism is a global health practice where patients travel abroad to receive health care. Voluntourism is a practice where physicians travel abroad to deliver health care. Both of these practices often entail travel from high income to low and middle income countries and both have been associated with possible negative impacts. In this paper, we explore the social responsibilities of medical tourists and voluntourists to identify commonalities and distinctions that can be used to develop a wider understanding of social responsibility in global health care practices.

**Discussion:**

Social responsibility is a responsibility to promote the welfare of the communities to which one belongs or with which one interacts. Physicians stress their social responsibility to care for the welfare of their patients and their domestic communities. When physicians choose to travel to another county to provide medical care, this social responsibility is expanded to this new community. Patients too have a social responsibility to use their community's health resources efficiently and to promote the health of their community. When these patients choose to go abroad to receive medical care, this social responsibility applies to the new community as well. While voluntourists and medical tourists both see the scope of their social responsibilities expand by engaging in these global practices, the social responsibilities of physician voluntourists are much better defined than those of medical tourists. Guidelines for engaging in ethical voluntourism and training for voluntourists still need better development, but medical tourism as a practice should follow the lead of voluntourism by developing clearer norms for ethical medical tourism.

**Summary:**

Much can be learned by examining the social responsibilities of medical tourists and voluntourists when they engage in global health practices. While each group needs better guidance for engaging in responsible forms of these practices, patients are at a disadvantage in understanding the effects of medical tourism and organizing responses to these impacts. Members of the medical professions and the medical tourism industry must take responsibility for providing better guidance for medical tourists.

## Background

The concept of social responsibility has been influential in guiding professionals' conduct, including in business [1,2], law [3,4], and medicine [5,6]. We understand social responsibility to entail the claim that an individual or group of individuals has a moral responsibility to promote the welfare of the communities to which they belong or with which they interact [6,7]. For businesses, for example, corporate social responsibility is the claim that corporations have a responsibility to promote the welfare of the communities with which they do business, including providing a living wage to their employees, operating in an environmentally sustainable manner, and ensuring that some of their profits benefit community stakeholders [8]. Similarly, lawyers have not only a fiduciary responsibility to their clients, but also, as members of a profession, are obligated to engage in *pro bono *legal work that aids community members who are unable to pay for their services [9]. And for members of the medical profession, there have long been calls for physicians to look beyond the good of their own patients and act also to promote health within their communities [10].

Typically, calls for social responsibility focus on an obligation to promote domestic welfare. However, as individuals participate in more globally-oriented practices, the scope of the targets of their social responsibility expands. This phenomenon is evident in the corporate social responsibility literature focusing on multinational corporations [8,11]. For multinational corporations, their social responsibility is not discharged simply by benefiting the communities in which their corporate headquarters are located. Rather, they must also ensure that stakeholders in all of the communities in which they operate benefit from their operations and that these benefits are sustainable over the long term. In practical terms, this might mean that multinationals that outsource manufacturing from their home countries must ensure that they pay a living wage to foreign-based workers, refrain from polluting foreign communities, and spread some of their profits both at home and abroad [12,13].

In this article, we explore the social responsibility of the participants in two global health care practices: voluntourism (travel abroad by physicians to deliver medical care) and medical tourism (travel abroad by patients to receive medical care). The terms 'voluntourism' and 'medical tourism' can both be seen as pejorative and normatively loaded given the connotation that each involves a frivolous, touristic element. For this reason, for example, some members of the medical tourism industry prefer labels such as 'medical travel' or 'global health care'. We use the terms 'voluntourism' and 'medical tourism' here because they are widely recognized and used in the academic literature. We do not intend to imply by the use of these labels that either practice is inherently morally problematic or any other related negative value judgments. While both of these groups share many attributes [14], we understand them to be distinct phenomena practised by different groups. We specifically aim to explore the nature of the social responsibilities of these two groups and to draw together parallels and distinctions that can be used to assist with articulating wider trends regarding social responsibility in global health care practices. In doing this we extend the traditional professional-centric focus of the social responsibility literature to consider the types of responsibilities inherent in the practice of medical tourism for international patients. While medical tourists travel from both developed and developing countries and represent a diverse range of income levels [15], we focus on the social responsibilities of relatively wealthy patients from high income countries in order to draw a parallel between the relative privilege of these patients and that of physician voluntourists traveling from high income countries. As we argue below, the better defined social responsibility of physicians engaging in voluntourism holds lessons for the rapidly developing practice of medical tourism. To accomplish our aim, we first provide an overview of the global practices of voluntourism and medical tourism, and then move to articulate the social responsibilities of voluntourists and medical tourists separately, focusing on the basis for their social responsibility and the targets of this responsibility. We then offer a discussion that compares these two groups, looking for overlaps and distinctive elements in their social responsibilities.

### Global Health Care Mobilities: Introducing Voluntourism and Medical Tourism

Recent years have witnessed the emergence of new forms of global health care mobilities, and increased popularity of existing forms due to processes such as the development of a globalized economy, establishment of international and bi-lateral trade agreements, and expansion of the international travel industry [16-18]. Patient mobilities (the movement of patients across international borders for service use) and provider mobilities (the flow of health care providers across international borders for service delivery) are two important forms of international health care mobility. These mobilities take many forms and involve flows between an almost endless number of home countries and destination nations. Provider mobilities can include permanent health worker migration and short-term relocation to enhance skills through training abroad [16,19], while patient mobilities can include accessing arranged cross-border care through referral and obtaining emergency care while abroad [17,20]. In the remainder of this article we focus on two specific forms of patient and provider mobility. Physician voluntourism and patient medical tourism are international health care mobilities that are both characterized by temporally-limited time abroad and engagement in a minimum of two health care systems, either as a user or provider.

Global health disparities and inequitable access to health care in developing countries is an ongoing concern for many physicians. For instance, sub-Saharan Africa has close to 25% of the global disease burden but has only 3% of the global healthcare workforce [21]. Globalized processes have enabled physicians from around the world, and particularly from high income countries, to participate in humanitarian "medical missions" to developing countries to administer medical care as physician volunteers [22]. Physician participants in these missions see themselves as part of a long-standing humanitarian tradition in medicine of bringing desperately needed medical care to vulnerable communities in developing countries. The popularity of medical volunteering is on the rise, with over 500 medical mission organizations in the United States alone that help to organize over 6000 short-term missions to foreign countries [23]. Medical students are also enrolling in increasing numbers to participate as volunteers in global health initiatives during their training. Current figures suggest that close to 30% of graduating North American medical students have taken part in a global health project [23]. Yet, there is also growing concern around the lack of ethical guidelines supporting medical missions and volunteerism that has resulted in the labelling of these terms as "physician voluntourism", used pejoratively to describe volunteering as initiatives that can do more harm than good [24-27]. Nevertheless, those who continue to participate in this practice see it as a social responsibility and a form of global citizenship [28].

Medical tourism, on the other hand, takes place when patients leave the country in which they live to pursue non-emergency medical interventions abroad [20,29]. The care accessed abroad is not part of an established cross-border care arrangement (e.g., does not involve physician referral), and is typically paid for out-of-pocket [20]. Medical tourism is thought to be a popular option for patients on wait-lists for care in their home systems, who have no health insurance or are underinsured, or who are looking to access experimental or illegal treatments [18,30,31]. A number of developing countries, including India and Thailand, have become leaders in this international industry [32]. Unfortunately no reliable estimates exist regarding the number of people travelling abroad each year as medical tourists [29]. Despite this, estimates regularly project growth in the industry in the years to come [20]. With the growth of the industry have also come concerns regarding the impacts it is having on destinations, particularly within developing nations. An oft-repeated worry is that it will exacerbate health inequities in both the destination and home country for medical tourists [20,29]. In the destination country, if medical tourists drive demand for expensive services, they may price out poorer citizens, or at least create a second tier of medical care in those countries [33,34]. Medical tourism may shift services from preventive public health measures to less effective, and more expensive, clinical interventions [35]. The development of private clinics serving foreigners may also encourage the movement of trained physicians from the public to private sphere [33,35]. On the other hand, proponents of medical tourism note its potential to cross-subsidize health care in the public sphere [36], though some of these agreements have been violated in practice [37].

## Findings

### Physicians' Social Responsibility in Voluntourism

Physicians have long embraced a fiduciary duty to manage and protect the health of patients, over and above their own self-interest. This fiduciary relationship plays a foundational role in medicine, and is founded on principles such as fidelity, integrity, compassion, courage, altruism, and justice [38]. The concept of social responsibility is informed by these principles, and is one that enables physicians to develop a public trust, and a professional identity around what it means to be a Doctor in society. There is a sense that modern day medicine is failing to recognize its societal role [39] and failing to educate physicians to meet the health care needs of a diverse society [20,40]. This situation is problematic as a physician's social responsibility to protect public interest is not an option, but a fiduciary duty that is entrusted to each and every physician, individually and collectively. It is based on the understanding that illness affects an individual's capacity to function as a productive and contributing citizen, member of a family unit, and part of the socio-economic system. Health and health care, therefore, are regarded by many countries as concerns of society as a whole and not simply those who are ill. In the remainder of this section we explore the various dimensions of physicians' social responsibility and consider how they relate to their involvement in the global health care practice of voluntourism.

#### Physicians' Social Responsibility

One manifestation of physician's social responsibility is the obligation to respond to inequities in health and how health services are organized in their domestic communities. It requires physicians to be mindful of responsibilities beyond individualism, profit, and private interests. The first Code of Ethics, issued by the American Medical Association in 1847, defines the duties of physicians to their patients, to each other, and to the general public:

*As good citizens, it is the duty of physicians to be ever vigilant for the welfare of the community, and to bear their part in sustaining its institutions and burdens: they should also be ever ready to give counsel to the public in relation to matters especially appertaining to their profession *[41].

Physicians are called upon to safeguard health systems so that services are effective, efficient, equitable, and sustainable [42]. Social responsibility is not simply a matter of charity, but a moral commitment to the patient that has been developed over centuries within societies that have advanced the conception of medicine as a profession. Professional status cannot be claimed without public sanction [43-45]. For this reason, physicians are required to maintain very high levels of expertise and skillfulness, as well as virtuousness and trustworthiness.

The provision of health care as a social security measure within an organized social system dates back to early Egyptian and Greek civilizations where physicians were hired by the state to treat its citizens without charge [46]. In 1601, Britain passed the Elizabethan Poor Law, allowing for a general taxation system to ensure medical care for the poor and infirm; and during the latter part of the Industrial Revolution several social, professional and religious associations or guilds also contributed a set sum of money voluntarily toward a form of protection that could provide assistance to its members who became incapacitated due to illness [47]. These early initiatives established a precedent regarding physicians' involvement in maintaining the social, or common, good beyond simply caring for their patients.

The notion of health care as a common good, rooted in social and religious ideas of charity, beneficence and compassion, is now recognized within the broader context of distributive justice, and the growing sensitivity to the equitable distribution of health care [48]. Hence, in medicine there is a growing reaffirmation that physicians have an obligation to the individual patient as well as an enduring responsibility to the broader society [49], particularly when dealing with issues around resource allocation, the social determinants of health, and related inequities. To this effect, the World Health Organization suggests that physicians need to be mindful of medicine's social responsibility [50]. Hence, medical organizations are called to direct their education, research and service activities toward addressing the priority health concerns of each community, region and/or nation that they have a mandate to serve, and particularly the more vulnerable and marginalized segments of their populations [51].

#### Medical Voluntourism and Physician Social Responsibility

Though discussions of physicians' social responsibility tend to focus on their responsibility to domestic communities, many medical students and physicians choose also to participate in medical voluntourism abroad out of a sense of social responsibility [21,28,52,53]. Through the act of voluntourism, these physicians invest personal time and resources toward reducing global health inequities. However, the growth of medical voluntourism is also outpacing the development of physicians' social responsibilities toward communities abroad and ethical guidelines to ensure that vulnerable communities are not subjected to more harm than good. Concerns about the lack of guidance for voluntourists derive in part from ethical tensions that emerge when research projects are conducted by researchers from high income countries in developing countries [24]. While host country members appreciate some aspects of these volunteers' work, responses to voluntourism are mixed [54].

A short-term clinical stint in a developing country can be seen as nothing more than a glorified form of tourism wrapped in a veneer of altruism, with no sustainable benefits for receiving communities [55-57]. Medical student voluntourists have also been criticized for using vulnerable people in developing countries to practice clinical skills, enhance résumés, and provide opportunities for travel to far-away and exotic places. Shah and Wu [58] provide a compelling example of the possible negative outcomes of medical student voluntourism through sharing a student's reflection:

*After finishing my first year of medical school, I participated in a mission trip to Mexico. Before flying to Mexico, I was not given any cultural, medical, or other training, nor could I speak Spanish. Upon arriving, I was assigned to a clinic where there were hundreds of patients but only one physician. I remember vividly seeing a frail 11-year-old boy with polyuria, polydipsia and nocturia. My lack of medical training limited my differential. With only a scattered history and no other tests, I told him to limit caffeine intake and see if that helps. Thinking back, he could have had a urinary tract infection, any number of renal abnormalities, or worse, I sent him out without ruling out diabetic ketoacidosis. And while I was seeing patients by myself, other first year medical students were performing surgeries in the other clinics and later bragging about it*.

The bragging by these students highlights the danger of voluntourism serving the needs of the voluntourist rather than the community abroad. Providing health care in international settings without carefully thinking about patient safety, sustainability, cultural appropriateness, quality of care, and consultation with local healthcare providers, among other similar issues, threatens to run counter to rather than discharge physicians' social responsibility abroad. Although participation in global health initiatives has great potential to offer medical trainees and physicians the opportunity to discharge their social responsibility [24], the risk of undesirable impacts from voluntourism can outweigh these benefits [59]. Vulnerable communities can easily become a means to the volunteers' ends instead of serving first the community's identified needs and empowerment interests.

Voluntourism is also often criticized for taking an exclusively charity-based approach to the provision of medical care, rather than enabling an equal and collaborative partnership with communities for developing capacity to address the root causes of systemic social inequity and disparity [60]. Charity based activities are based on the "good Samaritan" concept - providing resources, time, knowledge, and clinical service to vulnerable people. This approach is not only difficult to sustain, it can also create a dependency relationship through 'band-aid' solutions that do not address the root problem of health disparities. This line of criticism of voluntourism parallels critiques centred on the common establishment of temporally-limited selective primary health care initiatives in developing nations through aid programs, where a more community-centred intervention is thought to be the creation of long-term comprehensive primary health care plans [61,62]. In relation to voluntourism, a sustainable and community-centred approach requires physicians to focus their efforts on understanding and working to change the structural or institutional factors that contribute to inequitable conditions.

The Association of American Medical Colleges' (AAMC's) offers four foundational ethical considerations prior to embarking on global health voluntourism: (1) ensuring high ethical and moral standards, (2) developing a social contract with the communities served, (3) subordinating self-interest to the interest of the communities served, and (4) ensure that core humanistic values (honesty and integrity, caring and compassion, altruism and empathy, respect for self and others) are at the forefront of all activities [23]. These ethical considerations point to a number of specific social responsibilities that physicians involved in voluntourism hold, such as ensuring that compassionate and respectful care is provided that meets the highest ethical and moral standards that the context allows for. What these guidelines lack are specific, concrete strategies for enacting ethical, socially responsible care. The 4Rs that were developed by Aboriginal leaders in Canada to guide researchers in working with their communities, which are summarized in Table 1, offer some suggestions for specific strategies [63].

**Table 1:** The 4Rs of Ethically Sound Research

Ethical Principle	Strategy
Respect	Valuing cultures' and communities' diverse knowledges regarding health matters and developing knowledge that contributes to communities' and cultures' health and wellbeing
Relevance	Ensuring that research (or practice) is relevant to the culture and community
Reciprocity	Incorporating a two-way process of knowledge exchange and learning, where all parties benefit from these opportunities and the development of relationships
Responsibility	Fostering empowerment through allowing for active participation and rigorous engagement by all parties

Source: Kirkness & Barnhardt 2001 [63]

Generally, socially responsible medical voluntourism is a collaborative process that considers the full participation of local communities, local healthcare workers, and local health authorities [54]. It complements principles of international solidarity and social capital within the context of civil society, where voluntourists act voluntarily and without seeking personal profit to share benefits.

### Patients' Social Responsibility in Medical Tourism

While patients do not form a professional group, with their own institutions, leadership, and codes of ethics like physicians, there have been claims that individual patients do have social responsibilities to their domestic communities. Much of the literature on patient responsibility has focused on the degree to which patients are responsible for their own health [64]. This literature seeks to determine the balance between personal responsibility for health and the responsibility of communities for the health of their individual members. There is, however, some discussion of the responsibilities of patients to their domestic communities and to their health care systems [65,66]. In the remainder of this section we articulate the hallmarks of patients' social responsibility and consider the specific types of responsibilities international patients hold when they engage in medical tourism.

#### Patients' Social Responsibility

Patients may have a sense of social responsibility due to having a sense of solidarity among the members of a community (e.g., other clinic users). Solidarity can represent a sense of togetherness and independence between individuals. Community members need not feel personally close or attached, but rather are part of a system that is valuable. These systems are made up of shared institutions, an example of which is a health care system. For individuals, this sense of solidarity implies not simply that the individual receives benefits from these institutions, but that she also contributes back in keeping with a value of reciprocity. In the context of solidarity around institutions that provide for the health of a community, "people should not be only passive recipients of services but should actively contribute to and try to avoid harming the system. This means that they should act responsibly when it comes to their health and that it is justified to expect this to a certain reasonable degree" [66]. Without reciprocity, shared institutions are unlikely to survive and the shared good will be lost. On this reading of personal responsibility, looking after one's own health and the efficient use of public health care resources can be understood as an expression of solidarity with community members. In addition to responsibilities for one's own health, the patient may also be said to have responsibilities to: (1) others, in the form of not harming others and meeting the health needs of those under one's guardianship; (2) the health care system, so that it may function fairly and efficiently and serve as many people as fairly as possible; and (3) the judicial authority, where patient responsibilities have been codified explicitly [65,67].

Under public health care schemes, patients have a responsibility to look after their own health for their own sake, but also as a social responsibility to the other contributors to the health system and to the health system itself. For example, the Romanow Report in Canada includes a proposed health covenant that lists a series of responsibilities for Canadians, including to "observe good health practices, and to promote and support the well-being of their families and communities" and "to use the system prudently, and to support the system through their actions and tax dollars" [68] (*p.50*). Similarly, the National Health Service (NHS) in Scotland distributed a pamphlet called *The NHS and You *[69] that details both the responsibilities of the NHS to its patients and the responsibilities of patients to the NHS. These responsibilities are clearly directed toward the wider community and the system itself, as they are ways that the patient can help "yourself, other patients, and NHS staff" [69] (*p. 15*). These responsibilities include: treating NHS staff considerately, keeping appointments and informing staff if an appointment must be cancelled, keeping contact information up to date, following medical advice, using emergency services appropriately, finishing any course of medications, and helping to stop the spread of infection. The pamphlet also discusses other ways to help promote health, including by donating blood, organs, and tissues and by volunteering with the NHS. These responsibilities are intended to allow the public system to operate more efficiently and better serve the whole community.

While the Canadian and Scottish examples above are non-binding, a Medicaid member agreement in the US state of West Virginia is binding on its members. Some of the responsibilities listed in this document are responsibilities to look after the patient's own health, though these responsibilities too can be construed as a social responsibility to use public resources efficiently. Other listed responsibilities are more clearly injunctions against inefficient use of public resources. These responsibilities include requirements to show up on time for appointments ("I will show up on time when I have my appointments" and "I will bring my children to their appointments on time"), the responsibility to facilitate contact with the Medicaid system ("I will let my medical home know when there has been a change in my address or phone number for myself or my children"), and the responsibility not to misuse emergency services ("I will use the hospital emergency room only for emergencies") [70]. Similarly, the state Medicare program in Kentucky includes the interlinked goals to "1) Stretch resources to most appropriately meet the needs of members; and 2) Encourage Medicaid members to be personally responsible for their own health care" [71]*(p.3)*. As with West Virginia, the Kentucky plan targets additional 'get healthy' benefits to persons who document participation in identified healthy practices.

The guidelines shared above have been rightfully criticized as potentially shifting burdens onto the most vulnerable members of society as Medicaid users in the US fall into the lowest income brackets [72,73]. These concerns can be addressed by noting that the patient's social responsibility is coupled with society's responsibility to provide for community health and limited by the patient's capacity for choice. That is, we can describe the responsibilities of society to patients, particularly for the social determinants of health, while at the same time acknowledging the role of personal conduct not only in personal health, but also in the functioning of one's health care system. This mutual responsibility for health admits of degrees just as an individual's ability to control her health varies depending on contextual factors, including her position in her social hierarchy [65].

#### Medical Tourism and Patient Social Responsibility

If medical tourists have a social responsibility to look to the efficient functioning of their own domestic health systems, then participation in medical tourism will extend this responsibility to the health systems of the destination countries to which they travel and develop new connections. Medical tourism for procedures that will serve to undermine health equity and the sustainability of the health system in destination countries is therefore a potential violation of the patient's social responsibility. Crucially, however, many of the worries about the negative impacts of medical tourism on destination countries are matters of conjecture rather than well-established fact [29]. Moreover, while many instances of medical tourism may exacerbate health inequities, it is not clear that all forms of medical tourism are fated to do so. Medical tourists who wish to engage in forms of medical tourism that do not cause these negative effects for destination countries, then, will be faced with severe difficulty in assessing the effects of their travel.

Medical tourism has also been associated with negative effects for the patient's home country in terms of lessening equitable access to care. As medical tourism allows relatively wealthy patients to opt out of treatment in their home health care systems, it may undermine political pressure for change as privileged patients are able to have their health care needs met abroad [74]. If so, less privileged patients who are less mobile will be left in a lower tier of care at home. For publically funded health care systems, the practice of paying out of pocket for necessary medical services can also help to encourage the privatization of health services at home, which may also undermine health equity [75,76]. As with the negative effects of medical tourism on destination countries, however, these concerns are mostly matters of conjecture. While the patient may have a social responsibility not to travel abroad for care if doing so will undermine efficiency and equity in her home system, she may not have the information necessary to judge whether becoming a medical tourist will encourage these effects.

Medical tourism can serve as a means for patients to secure care more cheaply and quickly than if they remain within their local health care systems. However, travelling abroad for care creates a series of risks for the patient and long-term costs for the patient's home health system [20]. Travel itself creates risks by hastening the pace of care and surgeries and by increasing the risk of deep vein thrombosis or other complications from long plane flights [77]. Travel for care abroad can result in negative health consequences if an experimental treatment results in complications for the patient or other side effects [78]. While care abroad, even in low and middle income countries, is often of very high quality, poor oversight of facilities in some countries can result in sub-standard care and therefore complications and the need for follow-up care for the patient [79]. Patients receiving care abroad may also bring infections back home with them, including the NDM1 'superbug' that has been linked to medical tourists [80]. Finally, many forms of treatment require extensive follow-up care even if the principle intervention is successful or completed without complications. If arrangements for follow-up care in the patient's home country have not been made, then recovery can be delayed, resulting in complications [79]. Similarly, difficulties in transferring medical records between home and destination countries can complicate follow-up care [81].

As a result of these risks for medical tourists, they may incur more extensive expenses for follow-up care than persons remaining within their home countries. Insofar as these patients have a social responsibility to look to the efficient functioning of their home health care systems, engaging in medical tourism can potentially constitute a failure to discharge this social responsibility. Such an efficiency-based responsibility has been codified in Germany, for example. There, patients are asked to respect "the clinical and cost effectiveness of services, which are only to be used insofar as necessary" [67]. These responsibilities will exist for both members of public systems like those in the UK, Canada, and Germany, public portions of highly privatized systems like Medicaid in the US, and even insurance holders in privatized systems who have a duty of solidarity to their fellow insurance pool members.

As with other patient social responsibilities, the responsibility to use health resources efficiently should not undermine fair access to care, should not fall disproportionately on disadvantaged populations, and must admit of degrees in reflection of the extent of individual choice over health care decisions [67]. As some patients engage in medical tourism for necessary care that they would not otherwise be able to afford or access, their decision to go abroad for care may not be a matter of choice. Moreover, patients may not be aware of the dangers associated with medical tourism or the requirements for follow-up care for their specific procedures [75,82]. More generally, discerning the effects of engaging in medical tourism is difficult even for highly informed patients given a lack of data on the effects of medical tourism [29] and most patients would likely not have access to this information even if it were available. Therefore, it is inappropriate to hold medical tourists socially responsible for specific negative effects of medical tourism under these conditions. This is because discharging one's social responsibility by using health resources efficiently and mitigating third party harms requires knowledge of the effects of personal and health care choices [66]. Therefore, a first step toward a call to greater social responsibility among medical tourists is not to blame them for the effects of engaging in this global health care practice but rather to educate them on the effects of medical tourism.

In terms of assigning social responsibility for medical tourism, it is useful to differentiate between travel for medically-necessary and elective treatments. While many medical tourists travel abroad for much needed hip replacements, cardiac surgeries, or eye surgeries, other treatments such as elective cosmetic surgery would not be considered medically necessary. While we can grant that there will be considerable grey area between the categories of medically necessary and purely elective treatments, the differences between these two kinds of treatment have implications for whether patients are discharging their social responsibilities. If a treatment is not medically necessary but does create harms for others, including contributions toward health inequities in the destination country and public expenses for follow-up care in the patient's home country, then she can reasonably be held responsible for these negative effects. Such steps have been taken elsewhere. In Germany, for example, co-payments are required of patients needing treatment as a result of a "non-medically indicated measure such as cosmetic surgery, a tattoo, or a piercing" [67](*p.1188*). Even for medically-necessary treatments, however, any determination of whether the medical tourist has failed in her social responsibility will depend on that patient's ability to assess potential harms to the destination country and the degree of the patient's control over the decision to engage in the elective surgery.

If patients engaging in medical tourism do have a social responsibility to restrict their participation in this practice, we must be sure that this responsibility does not fall disproportionately on the poor, uninsured, and other vulnerable groups who may be driven into travel abroad for medical care due to a lack of options at home. The danger is that talk of patient responsibility can be used to further burden the most disadvantaged members of a community [83]. Any determination of whether a proposed social responsibility for medical tourists would unfairly burden certain patients will require reference to the particular context in which the patient acts, including whether her home health care system is public or private, the environmental health burdens faced by the patient, her socio-economic position within her community, and individual factors that might undermine her ability to access healthcare. While it is difficult to say in general and without reference to the particular circumstances of a patient what the extent of a medical tourist's social responsibility is, the claim that we have defended here is that individuals face a social responsibility to their health systems to use these systems efficiently and to protect fair access to others. By choosing to access the health systems of other countries, medical tourists expand the scope of this social responsibility, entailing new responsibilities to not unduly burden their home health systems and also to use the health systems of other countries both fairly and efficiently.

## Discussion

**Figure 1:**
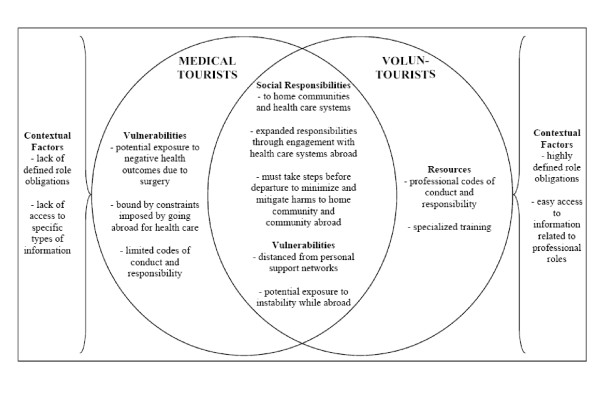
**Overlaps and Dissimilarities in Medical Tourists' and Voluntourists' Social Responsibilities**.

Voluntourism and medical tourism are both global health care practices that have dominant flows whereby citizens of the global north travel to the global south. As our discussion of both of these practices has shown, they each entail social responsibilities for their participants. By analyzing the similarities and points of divergence in the social responsibilities generated by voluntourism and medical tourism, we identify how our understanding of the social responsibilities of voluntourists can be illuminated by a discussion of the social responsibilities of medical tourists and vice versa. In Figure 1 we present a conceptual model that visualizes the similarities and points of divergence discussed in this section.

### Overlaps

Physicians and patients both have social responsibilities toward their domestic communities and health care systems. Physicians have an obligation to ensure that local medical systems are equitable and accessible and do not create conditions that encourage medical travel. As we have noted, physicians are bound by professional codes of ethics that require them to serve the interests of those in need. Physicians are in a unique position to meet the medical needs of their communities, and to refuse to do so can serve to show a callous disregard for these needs. Patients, we have argued, have a social responsibility to use medical resources responsibly and to take steps to avoid worsening the health of those around them, including through the spread of infectious diseases. A patient who took no steps to protect the health of fellow community members would, through her actions, not demonstrate respect for their claim to having their basic health needs met.

While voluntourists and medical tourists have social responsibilities to the communities with which they choose to engage, they are also put into positions of vulnerability by engaging in these practices of global health care that are undertaken across vast distances. Both voluntourism and medical tourism may entail travel far from one's home community. This travel may create stresses, including separation from one's friends and family, cultural and linguistic differences, and anxiety during the time abroad [20,22]. Voluntourists may face risks to their health and safety, particularly if they are traveling to a community that has poorly developed infrastructure, as will commonly be the case. Medical tourists are in a position of vulnerability as, like other patients, they face risks to their health from complications stemming from their medical procedures. But unlike most other patients, they often face these risks far from their support networks.

Persons engaging in voluntourism and medical tourism both can face exposure to political, social, and cultural instability. Voluntourists are called to administer care in communities abroad that are often impoverished, have poorly developed infrastructure, face political instability, and are exposed to endemic disease. While medical tourism is often advertised as providing patients with a safe and relaxing environment for care and recovery, they too can be exposed to unstable environments abroad. Many medical tourists were in Thailand during a recent outbreak of political instability, for example, and medical tourists may not be well informed about the local political conditions in the countries to which they are considering traveling [84]. Thus, both voluntourists and medical tourists, by choosing to travel abroad and engage in global health practices, are exposed to new vulnerabilities.

For both physicians and patients, the decision to travel to another country to receive or deliver health care serves to expand the range of the individual's social responsibility. The logic for this expansion of a pre-existing responsibility follows the rationale for the original social responsibility. Just as choosing to ignore the health needs of one's own community members when one could easily take steps to address these needs shows a disregard for others, engaging in voluntourism and medical tourism brings people into contact with new communities with their own distinctive needs. This contact creates new opportunities to take actions to meet local needs, or to ignore them altogether. Just as disregard for others' needs in one's original community would call into doubt one's commitment to others as having a right to adequate health, contact with a new community raises the possibility of similar, morally problematic inaction.

In order to ensure that they demonstrate concern for the needs of others and thereby discharge their social responsibilities, both voluntourists and medical tourists must take steps, before they travel abroad, to ensure that their choice to engage in these practices will not harm those with whom they come into contact. As we have observed, voluntourism raises the possibility of such harm if physicians fail to take into account the distinct needs of the local population, develop cultural and/or linguistic competency, or fail to ensure that the care they offer is sustainable. Medical tourists can encourage inequitable access to care in the countries to which they travel and may carry new infections to or create new costs for their home community. By taking steps to mitigate the potential for these harms prior to departure, voluntourists and medical tourists both help to discharge their social responsibilities.

### Dissimilarities

While both voluntourists and medical tourists face new vulnerabilities in virtue of their decision to travel abroad, the types and degrees of vulnerabilities faced by each group will likely be different. The key difference in these vulnerabilities is linked to the roles that each group takes when travelling abroad. The medical tourist often enters into travel in a very vulnerable position as she is seeking care to address her health needs. While some forms of medical tourism for purely elective procedures like cosmetic surgery may not place the medical tourist in a position of great need, any medical procedure carries risks of adverse side effects and post-operative infections. Some procedures, like cardiac surgery, will place the medical tourist in a position of great vulnerability due to high risk of negative outcomes [85,86].

While we should not discount the vulnerabilities faced by voluntourists, relative to medical tourists they will often be in a position of power due to the hierarchies implicit and explicit in the provision of medical care. The medical tourist may feel forced to travel abroad for care because of wait times for services or the high cost of medical care at home, particularly if the patient is uninsured [20]. The voluntourist, on the other hand, engages in this practice much more freely, though he or she may feel that doing so is part of an ethical obligation [7,44]. The knowledge and position of voluntourists allows them actively to provide medical services and intervene in addressing the needs of others. By contrast, medical tourists seek medical care and may be bound by a range of geographical and cost constraints.

The role of physician is much better defined than that of patient. While we have argued that patients are a group to whom distinct social responsibilities are attached, they are a more loosely defined group with fewer clear norms of behaviour and less of a governing institutional structure. While we all are patients at some points in our lives, physicians make up distinct professions, the membership of which is shaped by professional bodies. These bodies can in part dictate which individuals can be counted as members and help set governing norms for their behaviour. Thus, the social responsibilities of physicians, including those who choose to act as voluntourists, are much better defined than those of patients, who lack professional bodies to develop codes of conduct. Those codes of patient responsibility that we have identified and discussed above are typically the result of public health care institutions choosing to set norms for their members. Many patients, particularly in privatized systems, will not fall under the umbrellas of these public bodies, however, and will not be as clearly governed by these norms. Moreover, by choosing to travel abroad for care and, as is typical, pay out-of-pocket for this care, medical tourists frequently opt out of public health care systems and thereby the norms that dictate their responsibilities. For these reasons, codes of social responsibility for medical tourists have been slower to develop than those for voluntourists.

Finally, we have suggested that both voluntourists and medical tourists have a social responsibility to eliminate or mitigate any risks of harm to others that may be a consequence of their decision to engage in these global health practices. As we have already observed, medical tourists' choices may be much more circumscribed than that of voluntourists. Moreover, the information available to the medical tourists, with which they may attempt to mitigate the risk of harms stemming from their actions, is much more limited. As medical tourists are often very sick and in pain, they may not have the energy or focus to try to bridge these informational gaps. This informational asymmetry is due, in part, to the training of physicians compared to that of the typical medical tourist. Most of these international patients will not have access to specialized medical knowledge and may not be aware of the potential for medical tourism to exacerbate health inequities in destination countries or contribute to the spread of infectious disease. While physicians engaging in voluntourism will frequently receive specialized training specific to the context of the community to which they will be traveling, medical tourists typically travel at their own volition and without any formal guidance. Medical tourists may travel with the assistance of medical tourism facilitators [29], but we have no evidence that these facilitators provide information to medical tourists that would be relevant to discharging their social responsibilities. Thus, relative to voluntourists, medical tourists will often find it very difficult to determine how to mitigate any negative consequences of their travel, if they are even aware that such risks exist.

### Moving Forward

As we have discussed, physician voluntourism is seen a potentially ethically problematic approach to the provision of medical services in international settings, especially by students [87]. Hence, agencies that support medical student volunteers are beginning to insist on adequate pre-departure training to prepare them for the range of ethical issues they may encounter abroad [88]. Equipping volunteers for ethically responsible practices will require a transformative pedagogy [89], and the development of critical consciousness about the root causes of disparities in healthcare [90]. Pre-service medical training using international service-learning (ISL) opportunities appears to provide a promising experiential pedagogy for nurturing a sense of social responsibility and global citizenship among volunteers [24]. Unlike traditional voluntourism, ISL provides a platform for reciprocal, collaborative and mutual learning between a community and the volunteer. Volunteers are expected to develop a sense of critical awareness about the problems vulnerable communities face, and demonstrate ethical conduct and problem-solving skills as their experience in a given community unfolds. The focus of ISL is less on clinical skills development and more on developing an understanding of the social determinants of health that affect vulnerable communities. Interventions are designed in collaboration with communities in ways that are locally sustainable, enabling volunteers to learn how social determinants impact health and illness and health inequities [91].

Some training programs have successfully utilized the critical incident technique [92] to help physician volunteers explore how best to engage communities in ways that strive for social justice by understanding and acting to change the social structures that stifle individuals and communities due to unequal power relations, poverty and vulnerability. Physician volunteers are encouraged to develop a sense of professional and personal growth, and to examine critically what it means to be a socially responsible practitioner [93]. For example, many voluntourists seem to believe that being socially responsible means charity [60]. But charity can create dependency relationships whereas social responsibility aims at social justice, understood as developing sustainable relationships based on mutual respect. It involves working with and for communities to enable what they feel is best for them rather than using a paternalistic approach. Dickson and Dickson [60], identify a list of personal attributes that physicians need to develop as part of their professionalization and to act responsibly that include: a concern with global equity; a commitment to redressing injustices in healthcare; respect for diversity; openness to mutual learning; and embracing ethical values like human rights and social justice. The professionalization of physicians gives them norms by which their social responsibilities as voluntourists are increasingly clearly stated. It also gives physicians the information and expertise with which they may act on these norms.

By comparison, medical tourists are given little guidance on their social responsibilities and little capacity to act on these norms. In order to address this gap, there is great need for the development of guidelines for medical tourists on how they can prepare for their travel, engage in this practice with a minimum of personal risk, and take steps to maintain their own health and arrange follow-up care after travel. Because of the fragmented nature of the many community and national systems for distributing health care, these guidelines will likely take time to achieve widespread uptake. A starting point would be to have specific countries develop model patient guidelines that can be adapted and then adopted by other countries, thus encouraging better patient protections through their examples. These guidelines could be distributed by medical tourism facilitators or travel health providers. The latter group could be trained to give other advice on ethical medical tourism to their patients (Eyal: Global Health Impact Labels, submitted).

International patients need transparency so that they can make informed and responsible health choices. Given the lack of regulation in the medical tourism industry - due in part to its newness and global nature - patients may have difficulty getting information on the practices of the medical facilities they wish to travel to and may have no way of judging the accuracy of the information that they do receive [29]. One way of addressing this problem would be to develop ethical buying guidelines for patients engaging in medical tourism akin to those for consumers of other products like apparel, coffee, and chocolate. Patients will face difficulties in developing these guidelines on their own given the informational gaps discussed above and the fact that these patients may be in pain or short of time given their ill health. Moreover, the medical tourism industry may be reluctant or unable to develop these guides and to self-regulate both because of a reluctance to place restrictions on their business and because of the fragmentary nature of this industry at this early stage in its development. Non-governmental groups can take on the role of regulators, developing information from medical tourism facilities, assessing the accuracy of this information, and rating facilities for their tendency to promote the positive and mitigate the negative effects of medical tourism. While a non-governmental agency will not be able to compel medical tourism facilities to participate in a rating scheme, patients who see engaging in medical tourism responsibly as a moral obligation could create market pressure for participation (Eyal: Global Health Impact Labels, submitted). Alternately, patients' home country governments may need to take a more active role in regulating the medical tourism industry and providing patients with more information and transparency [94]. As medical tourism facilitators and other industry members can escape restrictions on their practices if they are developed piecemeal, there is a strong argument for finding ways to increase the information available to medical tourists and raising awareness of the potential negative effects of this practice. Doing so will allow medical tourists to apply pressure within the market for medical tourism services to counter problematic elements within this industry (Eyal: Global Health Impact Labels, submitted).

Medical professionals can and should help to develop the norms and infrastructure needed for medical tourists to discharge their social responsibilities. Doing so can be connected to these physicians' own responsibilities, as medical tourism will increasingly affect patients' home countries through the need for expensive follow-up care and the need to create systems to monitor and minimize the spread of infectious disease. Moreover, physicians can be implicated in the push factors encouraging medical tourism [20]. If patients are traveling abroad due to a lack of insurance, perceived lengthy wait times for care, or the unavailability of certain procedures, then health professionals must ask what their role is in reducing these factors and caring for the patients who choose to seek care abroad. While medical tourism and voluntourism are distinct global health practices, those engaging in them are closely connected through the increasingly global nature of health care, travel, and the business of medicine.

## Summary

Voluntourism and medical tourism share many qualities, including being tied to an expansion of social responsibilities due to an individual's choice to engage in a global health practice. Both voluntourists and medical tourists have a social responsibility to limit the risk of harms to members of their home and destination countries and to take steps to ensure that the global health practices in which they engage allow for sustainable development in their destinations. The complications associated with health care in low and middle income countries mean that voluntourists and medical tourists must prepare for their travel in order to avoid inadvertent harms to others. Yet, as we have seen, physician volunteers are much better prepared to do so than medical tourists in virtue of their membership in a well-organized professional group with a strong historical sense of social responsibility.

While physicians must continue developing and enforcing guidelines for discharging their social responsibility while practicing abroad, the field of medical tourism is less well prepared to develop the tools and guidelines needed for socially responsible medical tourism. As medical tourists themselves are not a well-organized group and may not be aware of the implications of their choice to engage in medical tourism, it is important that better organized and informed groups help to fill this vacuum. Physicians and other health professionals are members of groups that can draw on their knowledge, skills, and sense of social responsibility to help develop guidelines for responsible medical tourism. But it will also be important for medical tourism industry groups to engage in this process, including professional organizations like the Medical Tourism Association and medical tourism facilitators [95]. Fortunately, there is a long history of professional and business groups developing guidelines for socially responsible practice, from which medical tourism industry groups can learn [96]. It will be up for the rest of society, however, to help guide these groups and to ensure that they are encouraged or even required to develop these guidelines.

## Competing interests

The authors declare that they have no competing interests.

## Authors' contributions

JS wrote the introduction and summary, parts of the background, and the medical tourism sections of the discussion and edited throughout. SD wrote the social responsibility and voluntourism sections of the background and discussion. VC contributed to the background section, and contributed greatly to conceptualization and editing of this manuscript. All authors provided feedback on drafting this paper and approved the final version of the manuscript.
